# Keeping essential reproductive, maternal and child health services available during COVID-19 in Kenya, Mozambique, Uganda and Zimbabwe: analysis of early-pandemic policy guidelines

**DOI:** 10.1186/s12889-022-12851-4

**Published:** 2022-03-23

**Authors:** Marya K. Plotkin, Katie M. Williams, Absolom Mbinda, Vivaldo Nunes Oficiano, Benard Nyauchi, Patrick Walugembe, Emily Keyes, Barbara Rawlins, Donna McCarraher, Otto N. Chabikuli

**Affiliations:** 1 FHI 360, Durham, NC USA; 2 FHI 360 Zimbabwe, Mutare, Zimbabwe; 3 FHI 360 Mozambique, Nampula, Mozambique; 4 FHI 360 Kenya, Nairobi, Kenya; 5 FHI 360 Uganda, Kampala, Uganda; 6 USAID, Washington, DC USA

**Keywords:** Policy, COVID-19, Kenya, Mozambique, Uganda, Zimbabwe, Immunization, Antenatal, Contraceptives, Intrapartum, Postnatal

## Abstract

**Background:**

The COVID-19 pandemic has disrupted the provision of essential reproductive, maternal, newborn, and child health (RMNCH) services in sub-Saharan Africa to varying degrees. Original models estimated as many as 1,157,000 additional child and 56,700 maternal deaths globally due to health service interruptions. To reduce potential impacts to populations related to RMNCH service delivery, national governments in Kenya, Mozambique, Uganda, and Zimbabwe swiftly issued policy guidelines related to essential RMNCH services during COVID-19. The World Health Organization (WHO) issued recommendations to guide countries in preserving essential health services by June of 2020.

**Methods:**

We reviewed and extracted content related to family planning (FP), antenatal care (ANC), intrapartum and postpartum care and immunization in national policies from Kenya, Uganda, Mozambique, and Zimbabwe from March 2020 to February 2021, related to continuation of essential RMNCH services during the COVID-19 pandemic. Using a standardized tool, two to three analysts independently extracted content, and in-country experts reviewed outputs to verify observations. Findings were entered into NVivo software and categorized using pre-defined themes and codes. The content of each national policy guideline was compared to WHO guidance related to RMNCH essential services during COVID-19.

**Results:**

All four country policy guidelines considered ANC, intrapartum care, FP, and immunization to be essential services and issued policy guidance for continuation of these services. Guidelines were issued in April 2020 by Mozambique, Kenya, and Uganda, and in June 2020 by Zimbabwe. Many elements of WHO’s 2020 recommendations were included in country policies, with some notable exceptions. Each policy guideline was more detailed in some aspects than others — for example, Kenya’s guidelines were particularly detailed regarding FP service provision, while Uganda’s guidelines were explicit about immediate breastfeeding. All policy guidance documents contained a balance of measures to preserve essential RMNCH services while reducing COVID-19 transmission risk within these services.

**Conclusions:**

The national policy guidelines to preserve essential RMNCH services in these four countries reflected WHO recommendations, with some notable exceptions for ANC and birth companionship. Ongoing revision of country policy guidelines to adapt to changing pandemic conditions is recommended, as is further analysis of subnational-level policies.

## Background

Emerging empirical evidence along with modeling projections suggest that the COVID-19 pandemic is significantly impacting RMNCH services and health outcomes in many low- and middle-income countries (LMICs) [1]. Among reporting WHO member states in Africa, 57% experienced disruptions to immunization services [2]. One report describing the effects of COVID-19 on essential health services in eight LMICs documented a 10–24% decrease in routine immunizations in Burkina Faso, India, Kenya, and Nigeria; a 24–49% decrease in maternal and newborn health services in India; and a 50–74% decrease in family planning (FP) services in Pakistan [3]. Original modeling estimates indicated that, globally, up to 1,157,000 additional child and 56,700 additional maternal deaths could occur due to health services disruptions during the pandemic [4], and that an additional 49 million women could have unmet need for contraceptives, leading to an additional 15 million unintended pregnancies [5]. The COVID-19 pandemic has also been linked to rises in cholera [6], measles [7] and Ebola virus disease [8] (EVD) in sub-Saharan Africa (SSA).

Importantly, the majority of these additional deaths and unintended pregnancies was thought to be due to disruptions of health services [4]. During the 2014 Ebola virus disease (EVD) outbreak in West Africa, both immediate and sustained decreases in RMNCH service coverage were seen, with an overall 18% decrease in use of RMNCH services (including institutional births, FP, and antenatal care [ANC]) during the outbreak [9] — utilization did not rebound to pre-outbreak levels after outbreak control [10, 11].

The COVID-19 pandemic has placed massive pressure on health systems in Africa particularly [12]. For African countries already facing health system constraints — including lack of adequate financing, human resource shortages [13], lack of equipment, unreliable electricity supply, and supply chain disruptions [12–14] — COVID-19 represents an urgent challenge. African countries have substantial experience managing infectious disease outbreaks, including HIV, EVD, and tuberculosis [15]. However, health system gaps and environmental constraints — including the difficulty of social distancing in crowded urban centers and lack of water for handwashing — raise real concerns about avoiding high levels of COVID-19-related morbidity and mortality [4, 12, 15]. And while the continent reported fewer cases than predicted in the early months of the pandemic [16], by early 2021 cases were increasing [17] and evidence emerged of weak testing and disease surveillance systems which were potentially underrepresenting actual COVID-19 prevalence and impacts [14, 18]. Additionally, the burden of HIV and COVID-19 coexisting in the same populations are becoming more apparent. The African continent is home to two out of every three people living with HIV globally [19].

While many countries in sub-Saharan Africa have achieved significant reductions in maternal and child mortality over the past decade, COVID-19 has threatened to slow this progress. To reach global mortality reduction targets established for the 2030 Sustainable Development Goals (SDGs), countries must continue to ensure that essential health services, information, and commodities reach women, newborns, infants, and children and their families, without unduly increasing the risks of COVID-19 transmission while doing so.

Different possible approaches have been identified to mitigate exposure to COVID-19 during health service interactions. In the context of HIV service delivery, WHO’s call for differentiated service delivery (DSD) [20] pre-dates the COVID-19 pandemic but has been extremely useful to envision adapted services in the COVID-19 era. DSD has resulted in policy adaptations at country level such as: bundling services to reduce interactions between people and the health system; using telemedicine, employing community-based distribution systems; and multimonth drug dispensing, among other approaches [21]. Some literature has emerged to recommend similar adaptations to contraceptive services [22]. However, much less has been described in the literature related to health system adaptations meant to sustain RMNCH services in LMICs.

To successfully adapt service delivery models to pandemic-related threats, changes to national policy guidelines are required. These changes provide health care providers and administrators with an operational and legal framework for essential RMNCH service provision. Some of the most relevant policy factors to be addressed during the COVID-19 pandemic include: health care provider shortages and absenteeism; supply chain for RMNCH commodities, including contraceptives; reluctance of clients to seek care because of perceived risk of COVID-19 infection; travel or transport restrictions; and economic difficulties of users of the health system [4, 5, 13, 23].

Starting in March 2020, policymakers in Kenya, Mozambique, Uganda and Zimbabwe as in so many other countries were tasked with continuing essential, life-saving RMNCH services in a way that was least likely to contribute to new COVID-19 infections among both health care providers and clients. This analysis systematically describes how these four countries balanced continuation of RMNCH services — specifically, ANC, FP, intrapartum and postnatal care (PNC) and immunization services — with prevention of COVID-19 transmission. The purpose of this analysis is to describe the key policy elements driving service delivery in Kenya, Mozambique, Uganda, and Zimbabwe, including comparing with examples from HIV’s differentiated service delivery (DSD) models. We also compare WHO recommendations to what was described in each of the four countries’ national policies. In addition to filling the gap in systematic analysis of RMNCH service policy, this article will also provide contextual information to help interpret trends in service delivery during the pandemic in these four countries.

## Methods

We conducted a systematic review and analysis of policy guideline content related to RMNCH service provision during the COVID-19 pandemic to elucidate key policy approaches across Kenya, Mozambique, Uganda, and Zimbabwe. These four countries were conveniently selected because in each country, a USAID-funded technical assistance program led by FHI 360 was supporting the government to improve RMNCH service quality.

The analysis catalogued policy adaptations to RMNCH services and linked the adaptations either to continuation of a service (i.e., continuing availability of the service, commodities, or information to users of the service) or prevention of COVID-19 transmission within service delivery. The analysis was conducted using national-level RMNCH policy guidelines issued specifically to address RMNCH service delivery — subnational-level RMNCH policy guidelines were not included.

### Selection of policy guidelines

**Fig. 1:**
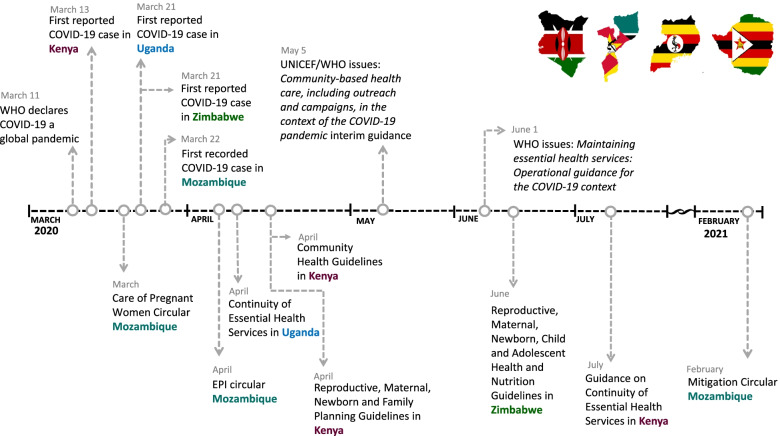
Timeline of COVID-19 policy developments

In each country, we formed a committee that included technical experts from USAID-funded RMNCH programs (Afya Uzazi in Kenya, Alcançar in Mozambique, Uganda MCHN Activity in Uganda, and Mhuri/Imuli in Zimbabwe). This committee proposed relevant policy guidelines for inclusion in the review and subsequently requested input from Ministry of Health (MOH) staff. Policies were included if they met two criteria: [1] issued by a national-level government agency in response to COVID-19 and [2] directly related to FP, ANC, intrapartum and postpartum care, and childhood immunization service delivery. After identifying policies that met the inclusion criteria, the expert committees added them to a repository of country policies. Only policies issued between January 2020 and February 2021 were included in the review (Fig. 1). Figure 1 also includes the release of two key policy guidelines issued by WHO and WHO/United Nations Childrens Fund (UNICEF).

### Data extraction and analysis

#### Content analysis using themes

Two analysts (KW, VN) used a standardized tool to extract content from the policy guidelines using pre-assigned categories, which fit under either “continuation of services” or “prevention of COVID-19 transmission.” The tool also had a category for “other” to allow for emerging in addition to pre-defined themes. Following the Grounded Theory approach [24], we included these emerging themes in the analysis. The analysts independently extracted data from each policy, compared extractions, and reached consensus about any conflicting interpretations. The completed data extraction form was verified by the committees before entry into NVivo software (Nvivo 12, QSR International) for analysis. Entry into NVivo was conducted by uploading documents which had both specific language from the policy guidelines as well as synthesis. An analyst (KW) went through the uploaded documents and tagged content using themes. Memos were produced which summarized the content under the themes. These data were reviewed by multiple analysts on the team.

#### Comparison to WHO recommendations on continuation of essential RMNCH services

Two analysts (MP, KW) reviewed WHO’s *Maintaining essential health services: operational guidance for the COVID-19 context* [25] of June 2020, particularly Part 2 on service delivery across life courses, which contains specific recommendations related to RMNCH services. Analysts selected recommendations from this section for their similarity to the findings derived from the content analysis. Related information from country policy guidelines was retrieved from the data abstraction sheets used for the content analysis. For each country results, the resulting information was sent to an in-country technical expert (AM, BN, PW, VN) who both reviewed and requested other national technical experts for review for accuracy. In Kenya, the information was verified with a MOH staff member in addition to the review by study team analysts.

### Ethical oversight

This study was reviewed by FHI 360’s Office of International Research Protection institutional review board and received a non-research determination. All policies reviewed were in the public domain. No people were interviewed as part of this study, so no informed consent was necessary.

## Results

The governments of Kenya, Mozambique, Uganda, and Zimbabwe all issued national-level policy guidance related to RMNCH service continuity (Table 1). Policies were formally issued in March (Mozambique), April (Kenya, Uganda), and June (Zimbabwe) of 2020. In Kenya, dozens of COVID-19 policy guidelines were issued by the MOH to cover various operational aspects of the health, transportation, and business sectors, and are available via a Kenyan government website [26]. Of these, three were directly applicable to RMNCH service delivery and thus included in this analysis. Mozambique issued two directives via circulars which were directly related to the analysis, one on maternal health and one on immunization, and a third circular was issued in February 2021 to broadly address mitigation measures for the health sector. In Uganda and Zimbabwe, respectively, guidelines were consolidated into one policy document.

**Table 1:** Policy guidelines included in the analysis

Country	Name of Policy Document (Naming Convention)	Issuing Authority	Date issued
Kenya	*Guidelines on Continued Provision of Community Health Services* (Kenya Community Health Services)	MOH, Division of Community Health Services	April 2020
	*A Kenya Practical Guide for Continuity of Reproductive, Maternal, Newborn and Family Planning Services in the Background of COVID-19 Pandemic*^a^ (Kenya RNMFP Guidelines)	MOH, Division of Reproductive and Maternal Health	April 2020
Mozambique	*Directrizes Operacionais para Cuidados com Mulheres Grávidas e Infecção pelo Novo Coronavírus* (Operational Guidelines for Care of Pregnant Women and Infection with the Novel Coronavirus) (Mozambique Care of Pregnant Women Circular)	Ministry of Health, Department of Public Health	March 2020
	*Orientaoes para o functionamento segura do Programa Alargado de Vacinacao em resposta ao COVID-19* (Circular: Guidelines for the Safe Operation of the Extended Vaccination Program (EPI) in Response to COVID-19) (Mozambique EPI Circular)	Ministry of Health, Department of Public Health	March 2020
	*Circular 01: Medidas de mitigação da COVID-19 no sector sáude* (Circular 01: COVID-19 Mitigation Efforts in the Health Sector) (Mozambique Mitigation Circular)	Ministry of Health, Department of Public Health	February 2021
Uganda	*Guidance on Continuity of Essential Health Services during the COVID-19 Outbreak* (Uganda Continuity of Essential Health Services)	MOH, General Health Services Division	April 2020
Zimbabwe	*Continuity of Reproductive, maternal, newborn, child and adolescent health and nutrition (RMNCAH-N) Essential Services in the COVID-19 Pandemic Context* (Zimbabwe RMNCAH-N Guidelines)	Ministry of Health and Child Care	June 2020

^a^This policy was updated in July 2020 in a document entitled, *Guidance on Continuity of Essential Health Services in the COVID-19 Pandemic*

**Fig. 2:**
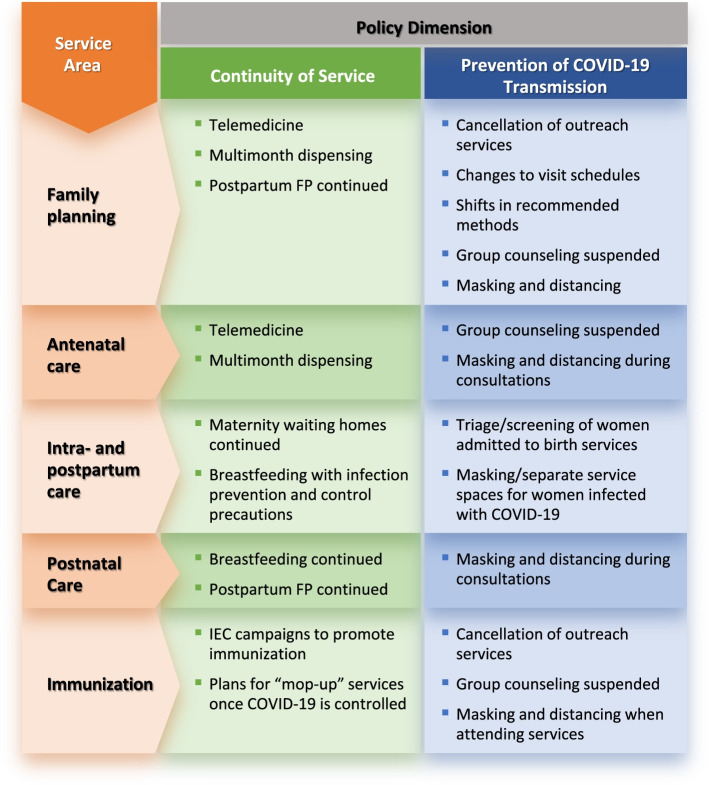
Summary of findings on RMNCH policy guidance, continuity of services, and prevention of COVID-19 transmission

The analysis grouped policy recommendations into two (overlapping) aims: continuing services and preventing COVID-19 transmission during provision of these services. Summarized findings from the four countries are presented in Fig. 2, in which recommendations related to continuity of services or prevention of transmission are divided by service areas (FP, ANC, intra- and postpartum care, and immunization). In brief, telemedicine and multi-month dispensing were recommended for FP and, in some cases, for ANC; all four countries specifically promoted immediate breastfeeding of newborns, with infection prevention and control (IPC) measures for the immediate postpartum period; and triaging and separating pregnant women infected or suspected to be infected with COVID-19 were described during the intrapartum period. Outreach services such as mass immunization campaigns were canceled for immunization services, and information, education, and communication (IEC) campaigns on the continuing importance of childhood vaccination were recommended in all four countries.

### Family planning

The key policy aspects supporting continuation of FP services included multimonth dispensing (generally for oral contraceptives, skin patch contraceptives, depot medroxyprogesterone acetate subcutaneous [DMPA-SC] in places where that method is available, and condoms) and telemedicine (in the form of consultations, reminders, or links to community health workers). Multimonth dispensing of short-term FP methods was described in Kenya, Uganda, and Zimbabwe (Table 2). In both Kenya and Zimbabwe, DMPA-SC was specifically mentioned, but not in Uganda or Mozambique. To prevent COVID-19 transmission, Kenya, Uganda, and Zimbabwe suspended outreach FP events or services and maintained other community-based distribution approaches. In policy guidelines for facility-based services, Kenya recommended immediately suspending group FP counseling. In Kenya, surgical voluntary sterilization methods including bilateral tubal ligation and vasectomy were suspended or postponed; in contrast, Mozambique, Uganda, and Zimbabwe policy guidelines specifically called for maintaining services for all contraceptive methods.

**Table 2:** Factors related to continuing services versus preventing COVID-19 in FP policy guidance

	Continuation of Services				Prevention of COVID-19 Transmission		
	Multimonth dispensing	Telemedicine	Outreach FP events or services	Community-based distribution of FP	Changes to facility-based services	Selected methods discouraged / temporarily suspended	Removal of long-acting and reversible contraceptives (LARCs)
Kenya RMNFP Guidelines	3-month supply of oral contraceptives (OCs), contraceptive skin patches	Tele-consults recommended for low-risk clients for condoms, OCs, and contraceptive skin patches	Outreach events suspended	Community-based distribution continues but limited to condoms and pills	Group counseling suspended immediately	Bi-tubal ligation and vasectomy services suspended or postponed	Removal of LARCs can be postponed as long as method within a safe period of expiration
Kenya Community Health Services	*Not mentioned*	Refills, counseling, SMS reminders to be provided by phone	*Not mentioned*	*Not mentioned*	*Not mentioned*	*Not mentioned*	*Not mentioned*
Mozambique Mitigation Circular	3-month supply for new users; 6-month supply for subsequent consultations	Telephone calls to be used for “as many services as possible”	*Not mentioned*	Home visits suspended	*Not mentioned*	No suspension of methods; all long-term methods to be offered at all times	*Not mentioned*
Uganda Continuity of Essential Health Services	3-month supply of OCs, DMPA-SC	Contacting client whose methods are expiring or village health workers (VHWs) to support them by phone	Outreach activities should be discontinued if evidence of community transmission	Community-based distribution should be maintained	*Not mentioned*	Provision of all modern contraceptives should be maintained	*Not mentioned*
Zimbabwe RMNCAH-N Guidelines	3-month supply of OCs	*Not mentioned*	Outreach services temporarily suspended, targeted outreach activities may be held at discretion of MOH	Outreach and related activities are suspended – Community based distributors or community health workers are to stay home and provide condoms only	*Not mentioned*	No methods are discouraged. Use of fertility awareness methods like standard days method or lactational amenorrhea method encouraged	Removal of LARCs conducted at facilities as possible; women who select unavailable methods to be contacted when these methods become available

In June 2020 *Maintaining essential health services: operational guidance for the COVID-19 context*, WHO makes recommendations on maintaining essential RMNCH services, including FP. All four countries included guidance which aligned with WHO’s recommendation of multimonth dispensing of FP (Table 3). Other WHO recommendations, such as making alternative contraceptive methods available in case a woman’s regular method is not available, were not mentioned in Mozambique and Uganda, but related messages were seen in Kenya and Zimbabwe. WHO’s recommendation on relaxing requirements for initial prescriptions for contraceptive methods was not mentioned in any of the four countries’ policy guidance. Only Uganda policy guidelines mentioned expanding availability of FP services to pharmacies and trained drug shops, as recommended by WHO.

**Table 3:** Selected WHO policy guidance recommendations^a^ for FP compared to country policy guidance

WHO Recommendation	Related Guidance in Country Policies			
	Kenya	Mozambique	Uganda	Zimbabwe
If a woman’s regular contraceptive method is not available, other contraceptive options should be made available (including barrier methods, fertility awareness-based methods, and emergency contraception [EC]).	For clients interested in surgical methods, alternatives to be offered (OCs, condoms, short-term injectable)	*Not mentioned*	*Not mentioned*	Clients interested in LARCs to be counselled on alternative methods when LARCs are not available
Relax requirements for a prescription for OCs, EC, or self-injectable contraception, and provide clear information for referral care for adverse reactions.	Condoms, combined OCs, combined contraceptive patches, and progesterone-only pills to be refilled without strict prescription requirement	*Not mentioned*	*Not mentioned*	*Not mentioned*
Provide multimonth supplies of contraceptives.	Recommends provision of 3-month supply of OCs, contraceptive skin patches	Recommends 3-month supply for new users; 6-month supply for subsequent consultations	Recommends 3-month supply of OCs, DMPA-SC	Recommends 3-month supply of OCs
Enable pharmacies/drug shops to increase range of contraceptive options; allow for multimonth prescriptions and self-administration of DMPA-SC if available.	*Not mentioned*	*Not mentioned*	Scale availability of FP services at all levels, including pharmacies and trained drug shops	*Not mentioned*

^a^*Maintaining essential health services: operational guidance for the COVID-19 context* (WHO, June 2020)

### Antenatal care

For continuation of ANC services, multimonth dispensing of ANC-related supplements and medications was not prominent. This approach was specified in policy guidelines in Kenya, but not mentioned in other country policy guidelines (Table 4). Telemedicine was addressed in Kenya in two ways: health care providers were advised to schedule phone-based ANC consultations as possible, and community health volunteers (CHVs) were provided messages that could be delivered via phone to pregnant women. In Mozambique, pregnant women with minor complaints were told to call the health care provider rather than coming to the health facility (“minor complaints” was not defined). Nothing about telemedicine for ANC was mentioned in Uganda’s policy guidelines. The current recommended schedule for ANC visits (eight visits for Kenya, Uganda, and Zimbabwe and four for Mozambique) was not changed, but in Kenya and Zimbabwe, it was recommended that some of these visits be phone consults rather than in-person visits to the health facility. Uganda guidelines did not mention the ANC visit schedule. Almost every reviewed country’s guidelines detailed physical distancing to be maintained during ANC consultations and mandated mask wearing; however, in the Kenyan RMNFP guidelines, distancing and masks are not specifically mentioned, but “IPC measures” were prescribed.

**Table 4:** Factors related to continuing services versus preventing COVID-19 in ANC policy guidance

	Continuation of Services			Prevention of COVID-19 Transmission	
	Multimonth dispensing	Telemedicine	8- or 4-visit schedule of ANC visits for pregnant women^a^	Physical distancing and other preventive measures during ANC services	Addressing COVID-19 driven stigma and/or psychosocial needs
Kenya RMNFP Guidelines	Women attending ANC be provided up to 3 months of supplements or medication, including antiretrovirals (ARVs)	ANC schedule to include phone-based consultations	The 8-visit schedule should be maintained, with modification of 4 health facility visits and 4 via phone if possible	ANC patients to visit clinics unaccompanied; telephone referrals and counseling encouraged	*Not mentioned*
Kenya Community Health Services	*Not mentioned*	A list of messages for VHWs to provide to pregnant women via phone consults. Guidance also promotes phone registration and follow-up of pregnant women.	*Not mentioned*	*Not mentioned*	*Not mentioned*
Mozambique Care of Pregnant Women Circular	*Not mentioned*	Pregnant women advised to call health care provider for minor complaints rather than visit health facility	Pregnant women to continue routine prenatal consultations (currently recommended at 4 focused ANC visits)	Keep distance of 1.5 m during facility ANC visits	*Not mentioned*
Uganda Continuity of Essential Health Services	*Not mentioned*	*Not mentioned*	*Not mentioned*	Ensure distancing (4 m) at ANC clinics	Psychosocial counseling and support to women with suspected / confirmed COVID-19 infection. Sensitize patients on mitigation of COVID-19-related stigma and discrimination and fear of mother-to-child transmission.
Zimbabwe RMNCAH-N Guidelines	*Not mentioned*	Phone consultations should be done as possible, either at nearby health facility or using a call center	8-visit schedule should be maintained; visit 3 (26 weeks) and visit 5 (34 weeks) should be conducted via phone (this may vary by level of health facility)	Ensure distancing, especially during triage at clinic arrival	Psychosocial support provided to women to reduce COVID-19 anxiety and fear, and to explain the changes in care management during COVID-19

^a^All countries have adopted the WHO-recommended eight-visit schedule except for Mozambique, which recommends an ANC visit every 3 months

A number of policy recommendations for ANC services by WHO [25] were also addressed in country policy guidance, while others were not (Table 5). WHO’s recommendations for prioritizing ANC services for third trimester clients and women with high-risk pregnancies were not mentioned in any reviewed country policy guidance, nor was discussion of adapting birth preparedness/complication readiness plans to COVID-19 health services, or booking ANC visits. Planning to provide all ANC services in a single service visit was similarly not included in any country policy guideline. Multimonth dispensing was mentioned only in Kenyan policy guidance, and this was limited to micronutrient supplements; insecticide treated nets (ITNs) were mentioned. The WHO recommendation most seen in country policies was that of using digital platforms for counseling and screening in ANC; related recommendations were seen in all country policy guidelines except Uganda’s.

**Table 5:** Selected WHO policy guidance recommendations^a^ for ANC compared to country policy guidance

WHO Recommendation	Related Guidance in Country Policies			
	Kenya	Mozambique	Uganda	Zimbabwe
Prioritize ANC services for women in third trimester and high-risk women	*Not mentioned*	*Not mentioned*	*Not mentioned*	Facilities to keep register of high-risk ANC clients for follow up via telephone
Ensure birth preparedness / complication readiness plans are adapted to COVID-19 services	*Not mentioned*	*Not mentioned*	*Not mentioned*	*Not mentioned*
Offer 2–3 months of recommended micronutrient supplements and ITNs	Women attending ANC should be provided up to 3 months of supplements or medication, including ARVs (ITNs not mentioned)	*Not mentioned*	*Not mentioned*	*Not mentioned*
Where feasible, use digital platforms for counseling and screening	ANC schedule to include phone-based consultations	Pregnant women advised to call health care provider for minor complaints rather than visit health facility	*Not mentioned*	Phone consultations should be done as possible, either at nearby health facility or using a call center
Where feasible, book ANC visits	*Not mentioned*	*Not mentioned*	*Not mentioned*	*Not mentioned*
Plan to provide all relevant ANC care in a single visit	*Not mentioned*	*Not mentioned*	*Not mentioned*	*Not mentioned*
Prioritize risk assessments for conditions known to be increased in COVID-19 context (tobacco, alcohol, other substance use; anxiety and depression; and gender-based violence [GBV])	Conduct screening for GBV (other risk assessments not mentioned)	*Not mentioned*	*Not mentioned*	*Not mentioned*

^a^ Maintaining essential health services: operational guidance for the COVID-19 context (WHO, June 2020)

### Intrapartum and postpartum care

Most of the elements described for intra- and postpartum care were related to continuity of services (Table 6). Policy guidance described whether birth companions could be present during birth (not allowed in Kenya, Mozambique and Zimbabwe; not mentioned in Uganda). All four countries’ policy guidelines encouraged immediate breastfeeding by all women, using precautions, including the mother wearing a mask and washing hands and breasts before breastfeeding. Zimbabwe was the only country that issued policy guidance about maternity waiting homes, specifying that they should be kept open. Postpartum FP was mentioned in policy guidance only in Kenya, which clarified that postpartum FP should be offered at all health facilities during the pandemic. PNC services, typically provided at either health facilities or via a home visit, were mentioned in all four countries’ policy guidance. In Kenya, the 2- and 6-week PNC visits were preserved and occur at a health facility, with recommendations on where a woman should seek care based on her risk category. Kenya’s guidance for CHVs included details of what could be discussed by phone regarding PNC. In Zimbabwe and Mozambique, policy guidelines specified that PNC visits should be provided with no modifications during the COVID-19 pandemic, whereas in Uganda, home PNC visits were to be suspended. Due to the nature of intrapartum care being necessarily facility-based, policy guidance to prevent COVID-19 transmission in intrapartum care from all countries focused on triage procedures during admission to maternity services or during intrapartum care, including screening women in labor for symptoms of or possible exposure to COVID-19 (all four countries), separate entry areas for those not yet screened (Uganda and Zimbabwe), and psychological support for women with COVID-19 (Zimbabwe).

**Table 6:** Factors related to continuing services versus preventing COVID-19 in intra- and postpartum care policy guidance

	Continuation of Services					Prevention of COVID-19 Transmission
	Birth companions	Immediate breastfeeding (BF)	Maternity waiting homes	Postpartum family planning	PNC	Triage procedures for women admitted for birth
Kenya RMNFP Guidelines	Birthing partners or companions discouraged from entering the intrapartum area	Immediate BF advised and preferred; COVID-19 confirmed or suspected women to be counseled	*Not mentioned*	Postpartum FP (and postabortion FP) will continue to be offered in all health facilities currently providing	2- and 6- weeks postpartum evaluation should be conducted. Women in a low-risk category should attend PNC at lower-level facilities, while women in high risk or who had cesarean section should be seen at Comprehensive Emergency Obstetric and Newborn Care (CEmONC) facilities	Screening for COVID-19 symptoms and exposure to someone with COVID-19 should be conducted before woman enter labor and delivery (L&D) room, and every 12 h after
Kenya Community Health Services	Annex on phone-based guidance for advising expectant mothers includes discussion of a support person/birth companion	*Not mentioned*	*Not mentioned*	*Not mentioned*	Annex on phone-based guidance for advising expectant mothers includes discussion of postnatal follow-up	*Not mentioned*
Mozambique Care of Pregnant Women Circular	Does not allow for the presence of birth companion during labor	BF encouraged with handwashing, masking, avoiding coughing or sneezing, cleaning surfaces	*Not mentioned*	*Not mentioned*	PNC recommended; specific guidance provided for postnatal follow-up with COVID-positive women	Women to be screened for COVID-19 symptoms upon arrival at health facility
Mozambique Mitigation Measures Circular	Prohibited for the duration of the pandemic	Promotion and counseling continued, exclusive BF recommended	*Not mentioned*	*Not mentioned*	Suspension of all home visits	*Not mentioned*
Uganda Continuity of Essential Health Services	*Not mentioned*	BF encouraged with handwashing, mask recommended; counseling on safe BF practices	*Not mentioned*	*Not mentioned*	Home PNC visits to mothers and newborns are temporarily suspended	Triage procedures for all patients, separate and dedicated entry to be established for “critical outpatient visits” including ANC
Zimbabwe RMNCAH-N Guidelines	Birth companions are discouraged during labor and delivery	Immediate BF encouraged for all, initiated 1 h after birth with hand and breast washing, mask wearing, physical distancing from others while feeding	Maternity waiting homes to be kept open	*Not mentioned*	PNC home visits to be conducted by VHWs; focus on BF promotion and neonatal and maternal health education	COVID-19 symptom screening for all patients, separate admitting area for suspected COVID-19 cases, different algorithms for different levels of health facility. Psychosocial support to be provided to L&D clients.

When comparing WHO’s guidance [25] to country policy guidance, only Zimbabwe’s policy guidelines mentioned that maternity waiting homes should be kept open (Table 7). WHO guidance promotes birth companions, subject to screening for COVID-19, while policy guidelines in Kenya, Mozambique and Zimbabwe stated that birth companions would not be allowed in birthing areas (not mentioned in Uganda). In line with WHO recommendations, all four countries specifically promoted immediate breastfeeding of newborns, with prevention measures that included washing hands and breasts, and each mother wearing a mask; however, none of the country policy guidelines mentioned skin-to-skin placement of the newborns, which was mentioned in the WHO recommendations. The only country policy guidance which mentioned safe transport of a pregnant women to the health facility, as was recommended by WHO, was Uganda, which described contacting *boda boda* motorcyclists for transport to facilities, including creating rosters and securing emergency travel permits for riders. Regarding cesarean deliveries, WHO recommends keeping the decision to conduct a cesarean delivery separate from COVID-19 exposure, infection, or transmission status. In Kenya and Mozambique, policy guidance stated to conduct cesarean deliveries as per pre-existing protocols, whereas in Uganda, the policy guidance specified that in the case of elective cesarean deliveries, a decision to delay could be made on a case-by-case basis. In Zimbabwe, the guidance appeared to contradict WHO recommendations, in that the guidelines named different levels of care and indicated that a delay of cesarean delivery may be possible for women suspected of having COVID-19 infection. WHO’s recommendation to provide PNC within 24 h was not specified in any of the four countries’ policy guidance. In Kenya, the guidelines specified that PNC follow-up visits should be made at 2 and 6 weeks, and for women with high-risk births, the determination should be made on a case-by-case basis. In Zimbabwe, follow-up of the new mother at home was advised to take place 7 days after delivery. Multiple recommendations from WHO on PNC were not mentioned in country policy guidelines, including using digital platforms for PNC, providing all relevant PNC services in a single visit, multimonth dispensing of supplements and medicines related to PNC, and updating PNC complication readiness plans to take into account changes to service provision.

**Table 7:** Selected WHO policy guidance recommendations^a^ for intra- and postpartum care compared to country policy guidance

WHO Recommendation	Related Guidance in Country Policies			
	Kenya	Mozambique	Uganda	Zimbabwe
Maintain maternity waiting homes where they exist, ensuring IPC standards	*Not mentioned*	*Not mentioned*	*Not mentioned*	Maternity waiting homes to be kept open
Screen birth companions for COVID-19	Birth companions not allowed during pandemic	Birth companions not allowed during pandemic	Birth companions not allowed during pandemic	Birth companions not allowed during pandemic
Ensure safe transport for mothers and newborns	*Not mentioned*	*Not mentioned*	At community level, coordinate *boda boda* cyclists for transport of pregnant women, newborns and children to facilities, especially in emergency situations. This may include mapping routes to facilities, creating rosters of *boda boda* riders, and securing emergency travel permits for riders.	*Not mentioned*
Prioritize skin-to-skin contact and early and exclusive breastfeeding	Promoted with infection prevention measures advised. Skin-to-skin contact not mentioned.	Promoted with infection prevention measures advised. Skin-to-skin contact not mentioned.	Promoted with infection prevention measures advised. Skin-to-skin contact not mentioned.	Promoted with infection prevention measures advised. Skin-to-skin contact not mentioned.
Cesarean section should be performed based solely on obstetric indications independent of COVID-19 status or transmission scenario	Deliver per pre-existing protocols	Caesarean section should be performed if indicated based on maternal and fetal status, as in normal practice	For elective cesarean procedures, case-by-case determination made to delay	At district, provincial, and tertiary levels, delay cesarean section for patients suspected to have COVID-19 to reduce risk associated with procedure (3 h for nulliparous, 2 h for multiparous)
Prioritize PNC contact with women and newborns during first week after birth, including contact within 24 h for home birth	Low-risk women with cesarean delivery review at 2 and 6 weeks; high-risk women determined individually	*Not mentioned*	*Not mentioned*	Follow-up by VHWs on day 7; no mention of contact within 24 h
Where feasible, use digital health platforms for PNC counseling and screening	*Not mentioned*	*Not mentioned*	*Not mentioned*	*Not mentioned*
Where in-person PNC visits are necessary, provide all relevant care in a single visit	*Not mentioned*	*Not mentioned*	*Not mentioned*	*Not mentioned*
At PNC, offer 2–3 months of micronutrient supplements, ITNs, and contraceptives as relevant, and consider offering LARCs	*Not mentioned*	*Not mentioned*	*Not mentioned*	*Not mentioned*
At PNC, ensure that complication readiness plans are adapted to take into account changes to services based on COVID-19	*Not mentioned*	*Not mentioned*	*Not mentioned*	*Not mentioned*

^a^ Maintaining essential health services: operational guidance for the COVID-19 context (WHO, June 2020)

### Immunization

Continuation of services featured prominently in the policy guidance recommendations for immunization (Table 8). One of the most significant considerations was maintaining or canceling outreach services. Zimbabwe was the only country whose guidance recommended continuing outreach services for immunization, specifying that outreaches should be smaller, held more frequently, and conducted outdoors. Outreach immunization services in Kenya were canceled in April 2020 guidelines; this temporary suspension was continued in an update to the guidelines issued in July 2020. According to policy guidelines of both Mozambique and Uganda, campaigns should be halted and immunization only offered at health facilities; Kenya guidance also recognized immunization as an essential service to be provided at the facility level. Immunization schedules are defined by both national and international guidance and changes in schedules are not advised in any country guidance. In Zimbabwe and Uganda, the policy mentioned “mop-up” campaigns to reach children who missed their appointments due to COVID-19. Mozambique, Uganda, and Zimbabwe policy guidelines all specified that communication campaigns should be used to promote the importance of childhood immunization and explain access to childhood immunization during COVID-19.

**Table 8:** Factors related to continuing services versus preventing COVID-19 in immunization policy guidance

	Continuity of Services		Prevention of COVID-19 Transmission
	Immunization “catch ups” after COVID-19	IEC communication campaigns	Outreach services
Kenya Community Health Services	VHWs to refer children who have not received scheduled vaccines to a health facility	*Not mentioned*	Immunization outreach services are suspended; immunization will be offered in health facilities only
Mozambique EPI Circular	Restructuring facility-based vaccination services to minimize opportunities for transmission. Integration of vaccinations into well-child visits to reduce missed opportunities.	Community radio shows will be used to share information on continuing need for child immunization and new health system approach to provision of the EPI program.	Immunization outreach suspended (mobile brigades, community campaigns); immunization services will be offered in health facilities only
Uganda Continuity of Essential Health Services	No change in the schedule of immunizations. Monitoring and contact tracing of children who miss vaccinations. “Mop-up” vaccination campaigns recommended post-COVID-19.	Radio and social media platforms to disseminate information about health service delivery	Mass vaccination campaigns are suspended
Zimbabwe RMNCAH-N Guidelines	Catch-up vaccination planned for children who missed vaccinations during lockdown period	Mass media programs to promote safety of vaccinations	Outreach efforts to continue, using guidance on the safe provision of services. Community campaigns may continue with smaller but more frequent sessions outdoors with high-risk populations.

WHO’s guidance [25] called for regular evaluation of evidence and surveillance data to inform how immunization services could continue to be offered, suggesting modifications that may help reduce risk of transmission within immunization services (Table 9). All country policy guidelines included some elements, such as improving IPC, while others were not seen in any country guidance (such as limiting the duration of visit to the health facility during immunization services).

**Table 9:** Selected WHO policy guidance recommendations^a^ for immunization compared to country policy guidance

WHO recommendation	Related Guidance in Country Policies			
	Kenya	Mozambique	Uganda	Zimbabwe
Train staff on IPC and delivery protocols	*Not mentioned*	*Not mentioned*	Health workers to follow IPC procedures during service provision	Health workers to follow IPC procedures during service provision
Provide facilities with adequate IPC equipment, including for waste management	*Not mentioned*	Personal protective equipment (PPE) provided, no mention of waste management	Use PPE, waste management procedures enforced	IPC equipment provided and waste management to be followed
Plan several small sessions per day at different times to limit contact	*Not mentioned*	*Not mentioned*	Immunize in groups no larger than five mother–child pairs	Schedule smaller, more frequent sessions; bundle immunization into other essential preventative health services
Limit the duration of stay in the health facility	*Not mentioned*	*Not mentioned*	*Not mentioned*	*Not mentioned*
Modify session locations to ensure separation of immunization services from treatment areas	*Not mentioned*	Outdoor or open spaces must be used for congregation, designated waiting areas	Use dedicated immunization clinics or separate spaces in health care facility	Use outdoor spaces and separate spaces in facility
Establish a screening process before allowing entry to the vaccination area	*Not mentioned*	*Not mentioned*	Triage clients and caregivers for symptoms and risk factors	Triage all clients entering health facility (not specific to vaccination area)
For outreach and mobile services, proactively engage with communities to identify open sites that allow physical distancing	*Not mentioned*	*Not mentioned*	*Not mentioned*	*Not mentioned*
Specific adaptations for birth doses and school-based vaccination^a^ [27]	*Not mentioned*	*Not mentioned*	School-based campaigns temporarily suspended	*Not mentioned*

^a^Maintaining essential health services: operational guidance for the COVID-19 context (WHO, June 2020) and Frequently Asked Questions (FAQ) about Immunization in the Context of COVID-19 Pandemic (UNICEF, 2020)

## Discussion

Soon after the COVID-19 pandemic was declared, policymakers in Kenya, Mozambique, Uganda, and Zimbabwe introduced changes to national policy to try to maintain RMNCH services in their countries. The urgency with which policy guidance was issued (in some cases, in less than a month following WHO’s announcement that COVID-19 was a global pandemic) reflects policy makers’ understanding of the immediate, life-changing and life-threatening impacts of disruptions to RMNCH services. Unavailability of contraceptives can quickly lead to unintended pregnancies; unavailability of condoms can immediately lead to new HIV infections; restrictions on access to skilled birth attendance can directly lead to maternal and newborn deaths. Our analysis describes country-level recommendations for FP, ANC, intrapartum and postpartum care, and immunization services during the COVID-19 pandemic, so that countries can learn from these policy responses as they face the ever-evolving challenges associated with COVID-19. Our analysis suggests that i) the RMNCH service package is complex; ii) gaps exist in original policy guidelines to varying extents in the four countries when compared to WHO guidance, and iii) policy guidance for RMNCH service delivery should be revisited given changing public health responses, mutations to the virus, vaccination availability, and new understanding of interactions between HIV and COVID-19.

### Key differences between country and WHO policy guidelines

As we have compared selected WHO recommendations for service delivery to the country policy guidelines, it is important to note that in Kenya, Mozambique, and Uganda, country policy guidelines were issued before the WHO guidelines, and in Zimbabwe, the policy guidelines were issued in the same month as WHO guidelines. The comparison is thus not meant to suggest deficiencies in content of country policy guidelines, but rather to highlight considerations for policy-makers as revisions are made to policy guidelines. In “normal” processes, WHO guidance comes first, then countries adapt the global guidance to local context, but during the COVID-19 public health emergency, this timing was altered [17]. While the four governments policy guidelines aligned with the majority of WHO recommendations, countries may wish to bolster their policies with WHO guidance in some service areas, notably allowing for birth companions, promoting skin-to-skin contact with the newborn, and multiple aspects of postnatal care. Following are some of key observations arising from the comparison between WHO and country policy guidelines.

#### Institutional births and birth companions

Globally, the majority of maternal and newborn deaths occur in the intrapartum period [28], and this is also the case in the four countries included in this survey. Because of this, birth with a skilled birth attendant (SBA) is high priority in all four countries - given current health structures, to have coverage by a SBA, births must necessarily occur in health facilities [29]. Birth companions are an important part of WHO’s respectful maternity care approach [30], and WHO COVID-related recommendations promote birth companions, subject to screening for COVID-19 [25]. All four counties have supportive policies for respectful maternity care, [31–33] but unfortunately, in all country guidance except for Uganda, having a birthing companion was halted. This may be an opportunity for WHO and the country policymakers to discuss how to preserve advances in respectful maternity care during the pandemic.

#### Immediate breastfeeding

One area of great alignment between WHO recommendations and country policy guidelines was the promotion of immediate breastfeeding of the newborn. All four countries’ policies specifically promoted immediate breastfeeding of newborns, with prevention measures to reduce risks of COVID-19 transmission. This has not been the case in all countries – case reports [34] as well as a Cochrane review of national policies in 19 countries [35] (including Asian and Latin American countries) found that in some countries’ early policy guidance, breastfeeding was contraindicated among mothers with confirmed or suspected COVID-19 or even asymptomatic mothers. Both modeling exercises [35] and discussion of mortality [34] and morbidity associated with COVID-19 exposure through maternal/newborn contact project far greater excess newborn (and child) mortality due to cessation of breastfeeding. Real life examples have emerged, as in the case in selected areas in India, of breastfeeding rates decreasing policy guidance on breastfeeding during COVID-19 is confusing [36]. While creating policies in an environment where there is little to no evidence is challenging, WHO and numerous experts, including International Planned Parent Federation [37], have urged policy makers to issue clear, supportive policy guidance to promote breastfeeding during the COVID-19 pandemic.

#### Outreach services

The cancellation of outreach services, most disruptive to immunization and FP services, is a very difficult decision for countries. Overall, 69% of WHO member states reported disruption to outreach services for immunization [2]. This dilemma was most reflected in immunization policy statements, as all four countries’ policy guidelines included explicit mention of resuming childhood immunization even as policies recommended canceling these services. The same urgency to resume outreach FP services was not as apparent in country policy guidance. This is concerning given one projected estimate of up to 15 million unintended pregnancies which may occur during the COVID-19 pandemic [38]. Uganda’s and Mozambique’s guidelines also specifically mentioned that the public needs to be informed of changes to service delivery, as WHO recommends in their statement, “During the disruptive event, communicating where, when and how the public can safely continue to access vaccination services is crucial.” [39]

### HIV, RMNCH and the need for integration of COVID-19 prevention

As the pandemic continues to unroll in Africa, the need for integration of COVID-19 vaccination for people living with HIV (PLHIV) is becoming more urgent, for multiple reasons. PLHIV in SSA appear to have heightened morbidity and mortality related to COVID-19, with one study showing up to four times higher likelihood of death among people with advanced or uncontrolled HIV [40]. Additionally, lower immune response may potentially lead to longer infection times, and eventually the development of COVID-19 variants of concern (VOC). Before the extreme spike in the Omicron variant of COVID-19 globally, a commentary in Nature warned of the link between prolonged infection among PLHIV and development of more highly transmissible variants [19].

Prior to the COVID-19 pandemic, the HIV community had introduced models of differentiated service delivery (DSD), which involves tailoring services to take into consideration clinical needs and characteristics affecting the population [17]. As the COVID-19 pandemic progressed, policymakers and implementing agencies provided guidance on myriad ways in which HIV service delivery can be modified, many of which focus on minimizing contact with the health system [41–43]. RMNCH service planners may glean some vital lessons from these efforts [44, 45]. Some DSD approaches to HIV services during COVID-19 include reducing frequency of visits, allowing peers to pick up medicines, allowing longer prescription periods and multimonth dispensing, decentralizing services to the private sector [46, 47], integrating HIV, hypertension and diabetes prevention and treatment services [45], moving services to outdoor spaces, shortening waiting times through “fast track clinics,” providing virtual services, and using the telephone to maintain peer support groups [17]. A “one-stop shop” model for testing mothers and providing services for early infant diagnosis, distribution of HIV self-testing kits during community mobilization events, and provision of “mother-baby packs” of ART and infant prophylaxis for women who test positive for HIV [9] are additional approaches for adapting services during COVID-19.

While differences between HIV and RMNCH are notable – for example, intrapartum care must take place in the health facility and is not a schedulable service - the principle of adapting services to account for peoples’ needs, environment, and circumstances is not only admirable, but also necessary to achieve continuity of essential RMNCH services. Policymakers are encouraged to incorporate “DSD thinking” into the policy and planning of RMNCH services, integrating RMNCH and HIV services, particularly in regard to reaching PLHIV with COVID-19 vaccines, and replicating the creativity and urgency with which the HIV community is adapting to mitigate the effects of COVID-19 on service delivery.

### Positive post-COVID impacts

While our analysis has revealed much disruption to RMNCH health services, a perspective is emerging that perhaps not all health system impacts of the pandemic will be negative. Some COVID-19 policy guidance may serve to improve efficiencies of health systems, translating into beneficial effects for clients [22, 48]. WHO’s June 2020 guidance on continuation of essential health services states: “As the outbreak is brought under control and restrictive public health measures are gradually eased, some adaptations in service delivery may need to be reversed, others continued for a limited time, and yet others that are found to be effective, safe and beneficial can be incorporated into routine post-pandemic practice.” [25] For example, the increase in telemedicine visits, in regions where the technology is largely available, may present a distinct advantage to clients accessing services. Not missing the opportunity to offer FP to clients who now make limited visits to health facilities, such as in the postpartum or postabortion period, has been cited as an opportunity to improve integration [48]. Multimonth dispensing of medications, which has accelerated dramatically in the health systems of low-, middle- and high-income countries alike, also increases efficiencies and may reduce health costs [25, 48].

The need for rapid mechanisms to adapt policy guidelines in the case of pandemics has been increasingly highlighted, with multiple mechanisms suggested, including increasing academic and government collaboration and creating multiple “templates” which could be rapidly applied [17]. A burgeoning number of resources are available to help countries assess evidence for and policy responses to COVID-19. For example, PATH, the Bill & Melinda Gates Foundation, and WHO collaborated on an online electronic policy database, which catalogues national and subnational policies related to continuity of essential health services from 119 countries [49].

The results of this analysis should be considered in light of the following limitations. The four countries were conveniently selected as countries in which the authors’ team had access to policy guidance and MOH colleagues to verify findings. Given the variation of content found in each country’s policy guidelines, findings may have limited applicability to other LMICs. However, we believe that our approach to the systematic review may be applicable to other country stakeholders who wish to apply an analytic lens to policy guidance on RMNCH services during the COVID-19 pandemic, particularly during policy revisions. Additionally, while our analysis only focused on national-level policies, we recognize that in many cases, subnational policy guidance may have had more immediate effect on service delivery. Indeed, colleagues in Kenya shared memos and circulars from county-level health authorities; these documents adjusted national-level policy to meet needs for immunization and other outreach services at the county level. We highly encourage more in-depth analyses that take subnational variations in policy into account.

Rollout of COVID-19 vaccines [50] and new strains of COVID-19 [51] necessitate revision of policy guidelines. Since the time these policy guidelines were released in 2020, breakthroughs in vaccine development, and subsequent massive vaccine inequity in SSA [52], have become pressing issues related to delivery of all health services across the continent. Similarly, the need for extensive improvements to disease surveillance in SSA countries has been identified [18]. While recognizing the need for continuing adaptation and improved systems, we offer the following suggestions for potential areas for improvement in national policy guidelines for continuation of essential RMNCH services:

- Provide more specific guidance on making telemedicine operational for RMNCH services. While all four country policy guidelines mentioned use of telemedicine for expanding access to health services, none of the reviewed policy guidelines contained instructions sufficient to guide health system administrators in how to use telephone technology to effectively deliver RMNCH services.
- Increase intention for multimonth dispensing in ANC. Multimonth dispensing for ANC medicines or supplements was only mentioned in Kenya’s policy guidelines. As per WHO recommendations, this is a recommended approach and an area of potential efficiency. Relevant approaches may include building upon suggested content areas and modes of service delivery for ANC mentioned in Zimbabwe’s policy guidance, which recommended visit content and mode of ANC service delivery by gestational age.
- Apply examples from DSD implemented for HIV services to rapidly learn how to tailor RMNCH services. While service platforms, content of services, and resource availability differ between HIV and RMNCH, the need for integration has become apparent, and both are served by the same health system. Existing analyses have started to draw lessons from HIV rollout to COVID-19 prevention services rollout [53]. Policymakers should continue this trend of learning lessons from HIV, accessing large volumes of published resources and in-country technical expertise.
- Revisit WHO’s recommendations to see where those not currently addressed within country guidelines may be applied. For example, allowing birth companions, which aligns with the countries’ own stated desire for improving respectful maternity care, may be revisited in areas where resources allow for screening for COVID-19.
- Bolster recommendations for PNC. In all four countries’ policy guidelines, PNC was not explicitly addressed, and in all four cases, the main recommendations by WHO for maintaining PNC were not seen in country guidelines

## Conclusion

In the first year of response to the COVID-19 pandemic, national policies from Kenya, Mozambique, Uganda, and Zimbabwe reflected a balance between the need to continue essential RMNCH services and the need to prevent transmission of COVID-19 in these services. For facility-based services that cannot be scheduled, such as intrapartum and immediate postpartum care, the focus was on triaging clients into categories of risk and setting up separated spaces. For services that included significant outreach components, the largest changes involved canceling outreach services and centralizing services at the health facility. Country policy makers will need to update RMNCH policy guidelines to adapt to vaccine availability and increased understanding of HIV and COVID-19, and incorporate global guidance from WHO during these revisions. Policymakers in these four countries are also urged to look to the DSD approach in HIV services for examples of how to adapt services to the needs of users during the COVID-19 pandemic, and to revisit WHO recommendations as they revise RMNCH policies to respond to the evolving conditions of the pandemic.

## Data Availability

The datasets generated and/or analyzed during the current study are available in the Harvard Dataverse repository, 10.7910/DVN/AGTUFE.

## Abbreviations

ANC: Antenatal care
ART: Antiretroviral therapy
CHW: Community health worker
COVID-19: Corona virus disease
DMPA-SC: Depot medroxyprogesterone acetate subcutaneous
DSD: Direct service delivery
EPI: Expanded Program on immunization
FP: Family planning
IEC: Information, education, communication
ITN: Insecticide-treated net
LARC: Long acting reversible contraceptive
MOH: Ministry of Health
OC: Oral contraceptives
PNC: Postnatal care
PPE: Personal protective equipment
RMNCH: Reproductive, maternal, newborn and child health
WHO: World Health Organization
